# A study on the development of happiness scale for Chinese young children

**DOI:** 10.3389/fpsyg.2024.1411480

**Published:** 2024-07-03

**Authors:** Jiaxin Xiang, Jieun Choi

**Affiliations:** ^1^School of Education, Guangzhou University, Guangzhou, China; ^2^University Innovation Support Project, Shinhan University, Gyeonggi-do, Republic of Korea

**Keywords:** Chinese, preschoolers, happiness, reliability, validity verification

## Abstract

This study was conducted to develop an assessment tool for measuring happiness among Chinese preschoolers, and to verify the reliability and validity of this tool. A total of 269 preschoolers aged 3 to 5 years from kindergartens and childcare centers in Hangzhou, China, were surveyed. The Preschooler Happiness Scale, constructed through literature review and interviews with preschoolers, underwent expert content validity verification and pilot testing to refine items. The validity and reliability of the scale were verified in this study, resulting in the development of the final Preschooler Happiness Scale comprising 6 sub-factors and 25 items. The reliability verification revealed a high overall reliability of 0.91 for the Preschooler Happiness Scale, confirming its trustworthiness as a tool. The academic significance of the findings and the practical utility of the Preschooler Happiness Scale in early childhood education settings in China were discussed based on the results of this study.

## 1 Introduction

The pursuit of happiness is not only a fundamental human right but also a topic of enduring interest among people. Preschoolers, like adults, desire happiness and experience various forms of it. Many researchers have suggested that the happiness experienced during early childhood can have a significant impact on life satisfaction and development even into adulthood (Lee and Seo, 2015). Individuals who had happy childhoods tend to develop emotional stability and empathy, leading to a higher likelihood of living a happy life in old age (Vaillant, 2010). Additionally, children who grow up feeling happy tend to be optimistic, intellectually active, confident, and socially competent, which in turn enhances the efficiency of all activities in life (Cho and Nam, 2011). Furthermore, happiness fosters adaptation, reduces disease incidence, and promotes both physical and mental health by fostering a positive outlook on life and the world (Jeon, 2011). Therefore, happiness is not merely a fleeting aspect of life after becoming an adult; it is considered a crucial factor that influences life quality and human development from an early age. Happiness can be developed by learning abilities such as cognition and creativity and by discovering and practicing one’s talents and characteristics (Seligman, 2004; Argyle, 2005). Hence, efforts to provide environments where children can experience happiness and enhance their sense of well-being from an early age are crucial for lifelong developmental purposes.

China recognizes the importance of a happy life, but despite rapid economic growth over the past half-century, many citizens are found to be unhappy with their lives, posing a serious social problem. According to the World Happiness Report by the UN in 2023, analyzing the happiness index of 156 countries worldwide, China ranked 60th (World Happiness Report, 2024). A study conducted by the Chinese Children’s Happy Growth Index Research Group on the Youth Happiness Growth Index surveyed 3,500 children in seven cities including Beijing and Guangzhou, revealing that 64.4% of the children reported being very happy, but 7.5% indicated that their happiness growth index had not yet reached the baseline (Chinese Children's Happy Growth Index Research Group, 2017). This indicates that these children lack happy experiences or are in a state of unhappiness for various reasons. A study analyzing the impact of different cultural contexts on the happiness of young children in China, the United States, and Korea found that, particularly in China, the harmony between traditional family values and modern educational methods plays a crucial role in the happiness of young children (Chen, 2012). A comparative study of students from four countries—China, South Korea, the United States, and Japan—showed that Chinese parents were more concerned about their children’s academic achievements compared to parents in other countries and were the least interested in their children’s emotions or friendships. However, emotions and friendships are major factors influencing a child’s happiness and are also areas where children are most likely to encounter problems (Sun, 2017). Additionally, it has been found that educational methods focusing solely on the infusion of pure knowledge determined by adults, while ignoring the mental needs of young children, are detrimental to their happiness (Feng and Liu, 2019). In response, a researcher argued through the broaden-and-build theory of positive emotions that positive emotions expand children’s cognitive and social abilities, and this expansion contributes to their happiness (Fredrickson, 2001). Thus, elements such as emotional stability, positive interpersonal relationships, and satisfying play experiences are included in the components of young children’s happiness, and these elements are closely related to their psychological well-being (Holder and Coleman, 2009). Research on happiness-related variables has revealed that factors such as children’s academic abilities, temperament, social skills, playfulness, and self-regulation abilities can influence their sense of happiness. Moreover, there is evidence to suggest that children’s happiness can reciprocally impact these variables, and studies have elucidated the correlations and mediating effects involved, indicating multidimensional research efforts (Lerner and Benson, 2013; Choi, 2022). Additionally, parental factors such as parental self-efficacy have been found to have a positive impact on children’s happiness, while variables related to maternal parenting stress and paternal involvement in caregiving have been shown to act as significant mediators. Furthermore, parenting styles, beliefs about play, and family health have also been identified as influential factors in children’s happiness (Sanders and Mazzucchelli, 2013; Cabrera et al., 2014).

With the emphasis on the importance of happiness in early childhood, the need for various educational programs, such as emotion coaching and social–emotional learning programs, has been suggested to enhance happiness during this critical period (Gonzalez-Mena, 2008). The Chinese Ministry of Education implemented the “Double Reduction” policy in 2021. This policy advocates spending joyful time by awakening children’s inherent nature and experiencing happiness through natural experiential activities from early childhood (Pi, 2012). The Ministry has not only advocated this theory but also implemented various activities aimed at promoting happiness using different media up to the present. However, when examining research on happiness in the field of early childhood education in China up to the present, studies have primarily focused on perceptions and surveys targeting young children or parents (Peng, 2008), as well as research on activities aimed at promoting children’s happiness (Zhou, 2021). However, the focus has largely been on the happiness of preschool teachers (Zhang, 2022; Huang et al., 2023). Despite the active research on happiness-related activities in the field of early childhood education in China, most existing happiness scales have targeted adolescents and adults. Scales developed for preschoolers either measure subjective happiness alone due to limited content (Wang and Yang, 2013) or focus solely on 5-year-olds (Qu, 2018).

Quantitatively measuring and evaluating the emotions and behaviors of young children is particularly complex. This is because it requires consideration of their developmental levels and characteristics (Denham et al., 2007). Moreover, the fact that many toddlers have limited language and communication skills adds another layer of difficulty to the development of happiness scales that take these factors into account. Moreover, existing measurement tools for preschoolers’ happiness primarily rely on self-reports (Wang and Yang, 2013; Abed et al., 2016; Qu, 2018), teacher assessments (Lee, 2010, 2016), or maternal assessments (Dehghani et al., 2016), neglecting the subjective and developmental nature of happiness. Therefore, there is a need for the development of reliable and valid tools that accurately measure preschoolers’ happiness levels, taking into account their perspectives and thoughts. Lyubomirsky et al. (2005) claimed that genetic factors account for 50% of happiness, while circumstances contribute 10%, and intentional activities contribute to 40%. The authors argued that although genetics and circumstances are beyond one’s control, anyone can achieve a happy life through intentional efforts. It is crucial to initiate intentional efforts to promote happiness from early childhood, as the happiness experienced during this developmental stage is likely to endure into adulthood. Therefore, programs and research aimed at enhancing children’s happiness should be activated. To achieve this, it is essential to develop reliable and valid measures that objectively and accurately assess children’s happiness levels. Thus, this study aims to develop a measurement tool for assessing the happiness of Chinese preschoolers aged 3–5 years and to validate its reliability and validity. The research questions based on the study objectives are as follows:

First, what are the final items of the Happiness Scale for Chinese preschoolers?

Second, what is the reliability and validity of the Happiness Scale for Chinese preschoolers?

## 2 Literature review

### 2.1 Happiness and its components

When conducting scientific research on happiness, one of the most important foundational tasks is defining the concept of happiness itself. In fact, defining “happiness” scientifically is as challenging as defining abstract concepts like “love.” Therefore, defining the happiness of children has become a major issue and research topic in happiness studies. To address this, researchers are making multidimensional efforts to operationalize the concept of happiness and measure it objectively. Children’s happiness is a state where they find satisfaction and pride in their daily activities and behaviors, including their relationships with parents, family, friends, teachers, hobbies, leisure activities, and play (Kim and Kim, 2008). Hwang et al. (2013) identify immersion as a subfactor of children’s happiness, which encompasses forming close relationships with family and friends, engaging in enjoyable activities, overcoming challenges, achieving goals, and experiencing joy. In this study, children’s happiness is defined as their self-assessment of overall quality of life according to their own standards (Diener, 1995). It consists of six subfactors: emotions, peer relationships, teacher relationships, family relationships, achievement, autonomy, and freedom.

### 2.2 The measurement methods of happiness

Currently, there are two main methods for measuring preschoolers’ happiness: structured surveys using questionnaires and open-ended inquiries such as the mosaic approach, narrative artwork analysis, and story completion tasks. Structured surveys primarily involve self-reporting by preschoolers (Kim and Kim, 2008; Wang and Yang, 2013; Jeon and Lim, 2014; Lee S, 2015; Abed et al., 2016; Qu, 2018; Jung and Shim, 2019) or assessments by others (Lee, 2010, 2016; Lee and Seo, 2015; Dehghani et al., 2016) in order to effectively collect data on preschoolers’ happiness. Happiness, being a highly subjective concept, is influenced more by individuals’ cognitive and evaluative judgments about their own lives than by external judgments (Diener, 1995; Seligman, 2011). Therefore, since personal perceptions and satisfaction with happiness are crucial, this study employs a self-reporting approach, where preschoolers’ happiness is assessed through their own stories and thoughts.

## 3 Methods

### 3.1 Participants

This study was conducted to develop an assessment tool for measuring happiness among Chinese preschoolers, and to verify the reliability and validity of this tool. For this purpose, a survey was conducted among 295 preschoolers attending kindergartens and childcare centers in Hangzhou, China. Out of the surveys distributed, a total of 274 were collected, and after excluding unreliable responses, 269 preschoolers were selected as the final participants for the study. Specifically, the participants included 86 children aged 3 (31.97%), 92 children aged 4 (34.20%), and 91children aged 5 (33.83%). The participants comprised 138 boys (51.30%) and 131 girls (48.70%).

### 3.2 The research procedure

**Figure 1:**
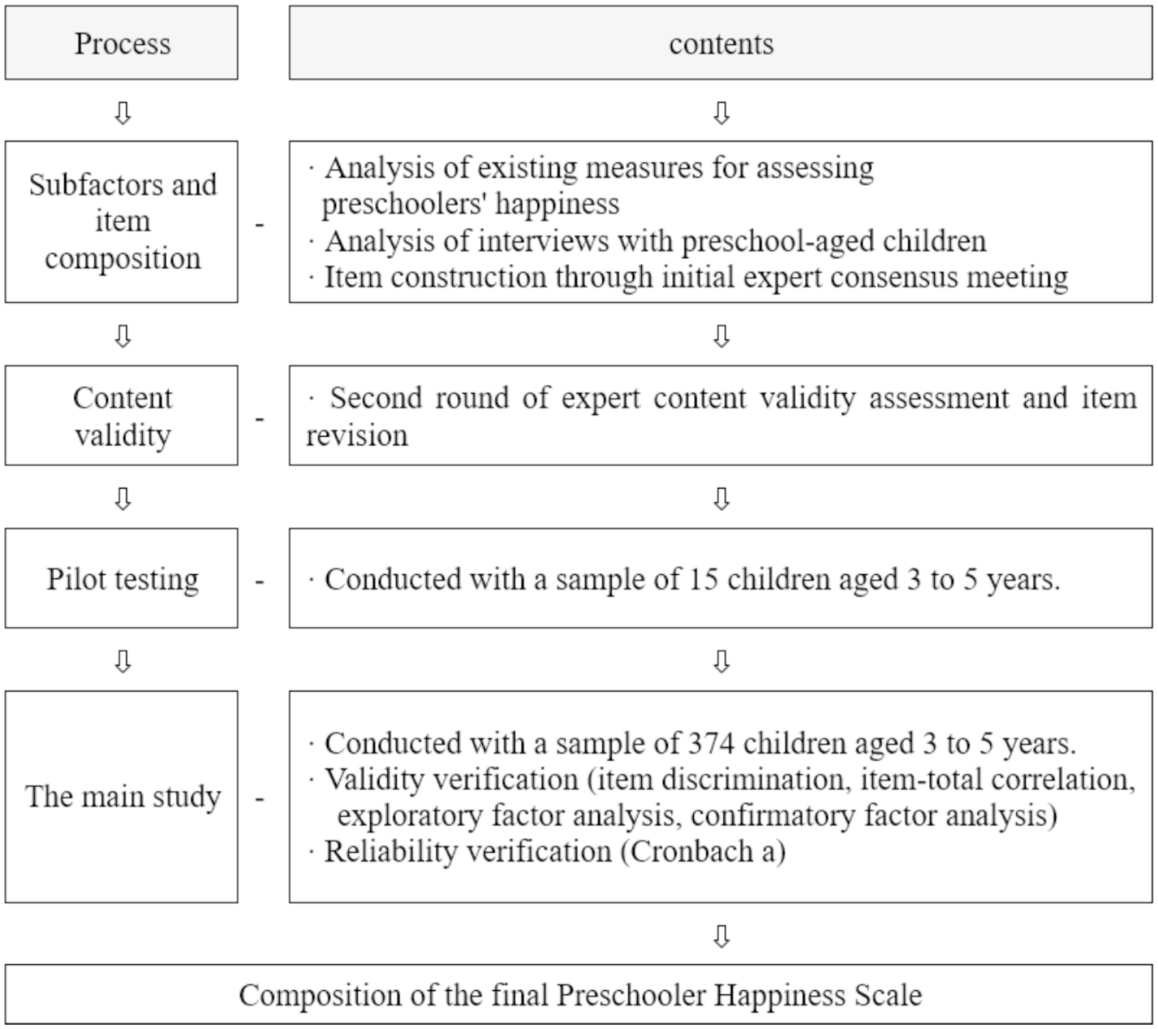
Development process of the preschooler happiness scale.

The items of the Preschooler Happiness Scale were constructed through literature review and interviews with preschoolers, followed by expert content validity verification and pilot testing to refine the items. Based on this, the validity and reliability of the scale were verified in this study, resulting in the development of the final Preschooler Happiness Scale. The process of developing the Preschooler Happiness Scale is depicted in Figure 1.

#### 3.2.1 Analysis of existing measures for assessing preschoolers’ happiness

Literature searches was conducted focusing primarily on concepts related to happiness, and well-being. Korean literature was searched using sources such as the National Assembly Library, the Korean Education & Research Information Service (KERIS), and the National Library of Korea. For Chinese literature, searches were conducted using platforms like CNKI (China National Knowledge Infrastructure), while for international literature, academic journals, theses, and dissertations were searched primarily using search engines such as EBSCO.

#### 3.2.2 Analysis of interviews with preschool-aged children

The researchers visited the participating kindergartens in advance to explain the purpose, content, procedures, and schedule of the study to the directors and teachers, obtaining prior consent for the research. The homeroom teachers distributed and collected research explanation sheets and consent forms to each household of the participating children. The final interviews were conducted with a total of 60 children, with 20 children from each age group (3, 4, and 5 years old), attending T Kindergarten in Hangzhou, China. The interviews consisted of two parts: “What is the happiest thing for you?” and “What is the least happy thing for you?” The interviews with the children were recorded and transcribed verbatim. The transcripts were coded sequentially as K1 to K60, and transcribed on the same day based on the recorded data and field notes taken by the researchers. The researchers paid attention to recurring words during the transcription process and analyzed the data.

#### 3.2.3 First expert consensus and item construction

The expert consensus was reached via an online conference using Tencent Meeting. Based on the analysis of existing literature on preschoolers’ happiness scales and analyzing interviews with preschool-aged children, the subfactors and items of the Preschooler Happiness Scale were developed through consensus among eight professors and PhDs in early childhood education.

#### 3.2.4 Second round of expert content validity assessment

The content validity verification was conducted through discussions between five professors in early childhood education and ten practicing teachers. Opinions regarding modifications to the items were collected through short-answer questions via the WJX survey platform. The appropriateness of the items was checked using a Likert 5-point scale ranging from ‘1 point – Not at all’ to ‘5 points – Very much’.

#### 3.2.5 Pilot testing

The pilot testing of the Preschooler Happiness Scale was conducted involving a sample of 15 children aged 3–5 years. Before conducting the pilot testing, the purpose and content of the study were explained to the directors and teachers of T Kindergarten in Hangzhou, China. In addition, informed consent forms and guidance documents detailing the purpose, methods, and content of the research were provided. After obtaining parental consent, the testing was conducted individually with each of the 15 children in a quiet and focused environment, using a one-on-one approach between the researcher and the child, within the counseling room of the kindergarten.

#### 3.2.6 The main study

The participants of this study were selected from children attending childcare centers and kindergartens in Hangzhou, China, excluding those who had participated in the pilot testing phase. First, prior to the administration of the study, research assistants received training to ensure accurate and smooth one-on-one interviews between the children and the examiners. During this session, the researchers provided detailed explanations about the purpose of the study, theoretical background, understanding of the Preschooler Happiness Scale, measurement methods, and important considerations during measurement. In addition, the examiners were given time to understand the examination process by watching recorded videos demonstrating how the researchers measured the happiness of children. Second, after explaining the purpose of the study to the directors and teachers of the childcare centers and kindergartens, their willingness to participate in the study was confirmed. Then, guidance documents outlining the purpose, methods, and content of the research, along with research consent forms, were sent to the parents. Third, a total of 269 parents agreed to participate in the study, and the researchers and research assistants conducted one-on-one interviews with the children using the Preschooler Happiness Scale. The measurement method involves teachers reading each item of the happiness scale to young children, who then use a Likert 5-point scale. Considering the children’s language abilities, they choose and indicate a card with five differently sized circles drawn on it, representing their self-reported level of happiness. The teachers read each item to the children, and if they do not understand, they assist them in understanding through specific situational explanations. After the children make their choice, the teachers ask why they chose that option, confirm their understanding, and then move on to the next item.

### 3.3 Data analysis

In this study, data were analyzed using SPSS 26.0 and AMOS 24.0 as follows. First, validity verification was conducted through item discrimination, item-total correlation, exploratory factor analysis, and confirmatory factor analysis on the collected data. Additionally, internal consistency reliability testing was performed for the items of the Preschooler Happiness Scale, and Cronbach’s alpha coefficients were calculated.

## 4 Results

### 4.1 Subfactors and item composition of the preschooler happiness scale

#### 4.1.1 Analysis of existing measures for assessing preschoolers’ happiness

Through a review of literature, existing measures for assessing preschoolers’ happiness were analyzed to identify the subfactors of preschoolers’ happiness. The analysis focused on papers that predominantly used measurement tools for assessing preschoolers’ happiness. The subfactors identified in existing measures for assessing preschoolers’ happiness were sorted based on frequency, as shown in Table 1. Commonly identified subfactors in existing measures included emotions, peer relationships, teacher relationships, family relationships, achievement, and life satisfaction. Additionally, factors such as health, engagement, self-esteem, and sense of safety were also identified as subfactors of preschoolers’ happiness. (A) Emotions: Existing measures for assessing preschoolers’ happiness identified emotions as a subfactor of preschoolers’ happiness (Lee, 2010, 2016; Wang and Yang, 2013; Lee and Seo, 2015; Lee S, 2015; Qu, 2018; Jung and Shim, 2019; Lee and Kim, 2020). (B) Peer Relationships: Existing measures for assessing preschoolers’ happiness identified peer relationships as a subfactor of preschoolers’ happiness (Kim and Kim, 2008; Lee, 2010, 2016; Jeon and Lim, 2014; Lee and Seo, 2015; Lee H, 2015; Qu, 2018; Lee and Kim, 2020). (C) Teacher Relationships: Existing measures for assessing preschoolers’ happiness identified teacher relationships as a subfactor of preschoolers’ happiness (Kim and Kim, 2008; Lee, 2010, 2016; Jeon and Lim, 2014; Lee and Seo, 2015; Lee H, 2015; Qu, 2018; Lee and Kim, 2020). D. Family Relationships: Existing measures for assessing preschoolers’ happiness identified family relationships as a subfactor of preschoolers’ happiness (Kim and Kim, 2008; Lee, 2010, 2016; Jeon and Lim, 2014; Lee and Seo, 2015; Lee H, 2015; Lee and Kim, 2020). (E) Achievement: Existing measures for assessing preschoolers’ happiness identified achievement as a subfactor of preschoolers’ happiness (Kim and Kim, 2008; Lee, 2010, 2016; Jeon and Lim, 2014; Qu, 2018; Lee and Kim, 2020). (F) Life Satisfaction: Existing measures for assessing preschoolers’ happiness identified life satisfaction as a subfactor of preschoolers’ happiness (Denham et al., 2007; Lee, 2010; Wang and Yang, 2013; Jeon and Lim, 2014; Abed et al., 2016; Qu, 2018).

**Table 1:** Analysis of existing measurement tools for preschoolers’ happiness.

		Researcher												
Subfactor		①	②	③	④	⑤	⑥	⑦	⑧	⑨	➉	⑪	⑫	⑬
Emotion					ㅇ	ㅇ	ㅇ		ㅇ	ㅇ	ㅇ	ㅇ	ㅇ	
Interpersonal relationships	Peer	ㅇ	ㅇ	ㅇ			ㅇ			ㅇ	ㅇ	ㅇ	ㅇ	
	Teacher		ㅇ							ㅇ	ㅇ	ㅇ	ㅇ	
	Family		ㅇ								ㅇ	ㅇ	ㅇ	
Ability/achievement		ㅇ		ㅇ						ㅇ	ㅇ		ㅇ	
Life satisfaction				ㅇ				ㅇ	ㅇ	ㅇ	ㅇ			
Health				ㅇ							ㅇ	ㅇ	ㅇ	
Engagement											ㅇ	ㅇ	ㅇ	
Home environment		ㅇ					ㅇ							ㅇ
Self-esteem					ㅇ	ㅇ								
Sense of safety				ㅇ									ㅇ	
Standards of living				ㅇ										
Self-identity		ㅇ												
Self-concept								ㅇ						
Self-acceptance							ㅇ							
Cognition														ㅇ
Spirituality											ㅇ			
Fulfillment of daily life needs			ㅇ											
Participation in helping activities			ㅇ											
Participation in play activities			ㅇ											
Psychological well-being														ㅇ
Sense of belonging				ㅇ										
Resilience								ㅇ						
Freedom										ㅇ				

① Kim and Kim (2008), ② Lee (2015a), ③ Jeon and Lim (2014), ④ Lee (2015b), ⑤ Jung and Shim (2019), ⑥ Lee and Kim (2020), ⑦ Abed et al. (2016), ⑧ Wang and Yang (2013), ⑨ Qu (2018), ⑩ Lee (2010), ⑪ Lee and Seo (2015), ⑫ Lee (2016), ⑬ Dehghani et al. (2016).

#### 4.1.2 Analysis of interviews with preschool-aged children

To explore the happiness perceived by preschoolers, qualitative research methods were employed through conducting interviews. The interview content consisted of questions such as “What makes you happiest?” and “What makes you unhappy?” Responses obtained from the interviews regarding what makes preschoolers happy, including peer relationships, teacher relationships, family relationships, achievements, autonomy and freedom, play activities, and fulfillment of needs. Preschoolers primarily reported feeling happy when playing games with friends, making new friends, receiving kindness from friends, receiving praise from teachers, having fun with teachers, playing with parents, achieving success, feeling autonomy and freedom, engaging in play activities, and having their needs met.

Regarding what makes preschoolers unhappy, responses were categorized as peer relationships, teacher relationships, family relationships, achievements, autonomy and freedom, and special events. Preschoolers expressed feeling unhappy primarily when experiencing conflicts with friends, being bullied, criticized by teachers, unable to be with or meet family members, criticized by parents, lacking autonomy and freedom, unable to achieve success, and encountering special events.

#### 4.1.3 1st expert consultation and item construction

Based on the analysis of existing measures of preschoolers’ happiness and interviews conducted with preschoolers, a panel of eight early childhood education professors and PhDs convened to establish sub-factors and items. Existing measures of preschoolers’ happiness commonly identified factors such as emotions, peer relationships, teacher relationships, family relationships, achievement, and life satisfaction. Similarly, the analysis of interviews with preschoolers highlighted factors such as peer relationships, teacher relationships, family relationships, achievement, autonomy, and freedom. Based on the analysis of existing measures and interviews, an initial version of the preschoolers’ happiness scale was developed following discussions with experts. The results of the initial expert panel indicated the inclusion of sub-factors such as emotions, peer relationships, teacher relationships, family relationships, achievement, and autonomy. Since life satisfaction, a sub-factor in existing measures, overlaps with factors such as teacher relationships and family relationships, it was removed, and its items were relocated to the sub-factors of teacher relationships and family relationships.

#### 4.1.4 2nd expert consultation

The content validity of the six subscales and 30 items developed through the initial expert consensus was further evaluated through a second round of expert consensus involving 15 early childhood education specialists. The content validity was assessed using a Likert 5-point scale ranging from ‘Not at all appropriate’ 1 point to ‘Highly appropriate’ 5 point. The content validity ratio (CVR) was calculated according to Lawshe’s (1975) formula, which is as follows:


$$CVR=\frac{N_{e}-N/2}{N/2}$$

*N* represents the total number of expert panelists, while *N_e_* refers to the number of expert panelists who rated the item as high (4 and 5 points). A higher CVR value indicates higher content validity of the item. According to Lawshe (1975), when there are 15 evaluators, a CVR value of at least 0.49 is considered necessary to ensure validity. Therefore, in this study, items 3 and 17 with CVR values below 0.49 were removed. Consequently, through the second round of expert consensus on content validity, a total of six subscales and 28 items were established.

### 4.2 Pilot testing

A pilot test was conducted to assess the comprehension of the happiness scale items among 15 children aged 3–5 years. Measurements were conducted through individual interviews between the researcher and each child in a quiet and focused kindergarten counseling room. The children demonstrated understanding of each item on the happiness scale and also comprehended the measurement method, which involved selecting and marking one of five differently sized circles drawn on cards. Therefore, it was confirmed that all 28 items and the measurement method were well understood by the children.

### 4.3 The main study

#### 4.3.1 The item discrimination and item-total correlation of the scale measuring children’s happiness

The item discrimination involved analyzing differences between upper and lower groups, with each group consisting of 27% based on item scores. Items with non-significant CR values (< 3) were deleted from the analysis. Consequently, all items of the Children’s Happiness Scale were retained as they met the criteria. Furthermore, correlation analysis was conducted between overall children’s happiness and each item. Items with item-total correlations below 0.30 were considered for deletion due to poor homogeneity. As shown in Table 2, all 28 items of the Children’s Happiness Scale demonstrated high homogeneity with item-total correlations exceeding 0.30.

**Table 3:** The model fit of the children’s happiness scale was assessed.

	*x* ^2^	*df*	*x*^2^ /*df*	RMSEA	GFI	AGFI	CFI	IFI	NFI	TLI
Model	302.70	260	1.16	0.03	0.92	0.90	0.98	0.98	0.91	0.98

#### 4.3.2 Exploratory factor analysis of the children’s happiness scale

Before conducting exploratory factor analysis to confirm the construct validity of the Children’s Happiness Scale, we assessed whether the collected data were suitable for factor analysis by performing the Kaiser-Meyer-Olkin (KMO) measure of sampling adequacy and Bartlett’s test of sphericity. A KMO value closer to 1 indicates high inter-correlation among the factors, with values above 0.70 indicating suitability for factor analysis and higher validity. Bartlett’s test assesses whether the observed variables intercorrelate significantly, with a significance level below 0.05 indicating that the data are suitable for factor analysis. The KMO value was 0.89, and Bartlett’s test of sphericity yielded a statistically significant result of 3898.48 (df = 378, *p* < 0.001). Thus, the collected data are suitable for factor analysis, indicating the presence of common factors.

As shown in Table 2, the results of exploratory factor analysis revealed that the overall explained variance of the Young Children’s Happiness Scale was 65.50%. Specifically, Factor 1, which accounted for 12.64% of the variance, consisted of 5 items. This factor evaluated both positive and negative emotions such as joy, sadness, boredom, loneliness, and anger, hence named “Emotion.” Factor 2, explaining 12.34% of the variance, included 4 items after excluding items 12 and 22, which showed correlations above 0.40 with multiple factors. This factor assessed children’s peer relationships and support received from peers, hence named “Peer Relations.” Factor 3, explaining 11.32% of the variance, consisted of 4 items. This factor evaluated children’s relationships with teachers and the support received from teachers, hence named “Teacher Relations.”

**Table 2:** Exploratory factor analysis of preschoolers’ happiness scale.

Item	Factors						Communality
	1	2	3	4	5	6	
T11	0.79						0.71
T10	0.75						0.72
T21	0.73						0.64
T1	0.72						0.57
T18	0.68						0.67
T19		0.82					0.78
T3		0.79					0.76
T20		0.74					0.65
T2		0.72					0.61
T12		0.62	0.60				0.77
T22		0.58	0.56				0.69
T23			0.78				0.74
T24			0.73				0.70
T5			0.70				0.68
T4			0.65				0.54
T25				0.80			0.69
T15				0.76			0.61
T8				0.75			0.70
T9				0.69			0.58
T16	0.41			0.46			0.41
T27					0.81		0.72
T6					0.79		0.67
T26					0.76		0.63
T17					0.72		0.57
T7						0.80	0.74
T14						0.78	0.68
T13						0.75	0.60
T28						0.72	0.61
Eigenvalue	3.54	3.46	3.17	2.85	2.71	2.62	
Explained variance	12.64	12.34	11.32	10.16	9.69	9.35	
Cumulative variance	12.64	24.98	36.30	46.46	56.15	65.50	

**Figure 2:**
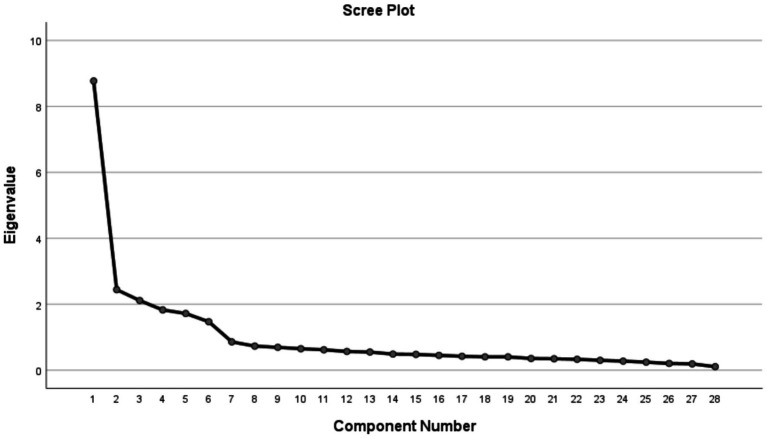
Results of the screen test for the scale of children’s happiness.

Factor 4 explains 10.16% of the variance and includes 4 items, excluding item 16, which shows correlations above 0.40 with other factors. This factor evaluates children’s abilities to do things well or have things they can do well, so it is named ‘Achievement’. Factor 5 explains 9.69% of the variance and includes 4 items. This factor evaluates children’s autonomy and freedom in their daily lives, so it is named ‘Autonomy and Freedom’. Factor 6 explains 9.35% of the variance and includes 4 items. This factor evaluates children’s family relationships and support from their family, so it is named ‘Family Relationships’. Therefore, the scale of children’s happiness was revised to 25 items by deleting items 12, 16, and 22. The results are depicted in Table 2 and Figure 2.

#### 4.3.3 Confirmatory factor analysis of the scale of children’s happiness

As shown in Table 3, to verify construct validity, confirmatory factor analysis was conducted, and the fit indices of the measurement model are as follows. Generally, when GFI, AGFI, CFI, IFI, NFI, and TLI values are above 0.90 and RMSEA value is below 0.05, it indicates a good fit of the model. The fit indices of the model for the developed Children’s Happiness Scale in this study are as follows: χ^2^ = 302.70(df = 260), RMSEA = 0.03, GFI = 0.92, AGFI = 0.90, CFI = 0.98, IFI = 0.98, NFI = 0.91, TLI = 0.98, indicating that all indices meet the recommended fit criteria.

**Figure 3:**
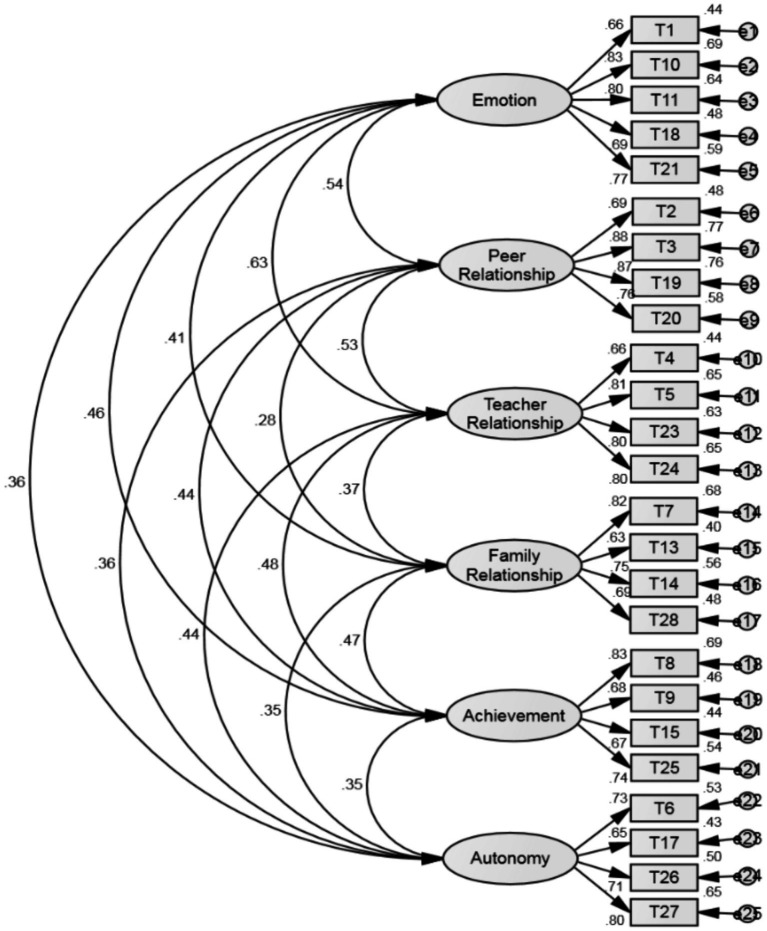
The structural model of children’s happiness scale.

Furthermore, to explain the relationships between variables in the children’s happiness model, parameter estimates were obtained using maximum likelihood estimation. The results are depicted in Figure 3.

#### 4.3.4 Convergent validity of the children’s happiness scale

Convergent validity was assessed by the Average Variance Extracted (AVE) and Construct Reliability (CR). AVE above 0.5 and CR above 0.7 were considered evidence of convergent validity. AVE was larger than 0.5 for both emotion (AVE = 0.57), Relationship of peer (AVE = 0.65), Relationship of teacher (AVE = 0.59), Relationship of family (AVE = 0.53), Achievement (AVE = 0.53) and Autonomy and Freedom (AVE = 0.53) attesting the convergent validity of all the first order constructs (Lazemi and Barkhordari-Sharifabad, 2023; Lu et al., 2023) (see Table 4).

**Table 4:** Convergent validity evidence of the scale.

Subfactor	Number of item	AVE	CR
Emotion	5	0.57	0.87
Peer relationships	4	0.65	0.88
Teacher relationships	4	0.59	0.85
Family relationships	4	0.53	0.82
Achievement	4	0.53	0.82
Autonomy and freedom	4	0.53	0.82

#### 4.3.5 Discriminant validity of the children’s happiness scale

Evidence of discriminant validity between first order constructs was assessed with the criterion of the Heterotrait-Monotrait ratio (HTMT). HTMT below 0.9 was considered evidence of discriminant validity (Sharif-Nia et al., 2023). According to the HTMT, more liberal criterion discriminant validity was observed between the 6 engagement constructs (see Table 5).

**Table 5:** Heterotrait-Monotrait ratio of discriminant validity evidence.

Heterotrait-Monotrait ratio (HTMT)	1	2	3	4	5	6
Emotion	–					
Peer relationships	0.53	–				
Teacher relationships	0.63	0.54	–			
Family relationships	0.42	0.29	0.36	–		
Achievement	0.46	0.45	0.47	0.46	–	
Autonomy and freedom	0.34	0.37	0.43	0.35	0.35	–

#### 4.3.6 Age-invariance analyses and sex-invariance analyses

Table 6 shows changes in CFI and RMSEA values lower than 0.010 and 0.015, respectively, between the successive constrained models for the 6 factor correlated structure. Therefore, the null hypothesis of invariance across gender and age cannot be rejected.

**Table 6:** Age-invariance analyses and sex-invariance.

Model	*x*^2^	*df*	*P*	CFI	RMSEA	Δ*x*^2^	*Δdf*	Δ*P*	ΔCFI	ΔRMSEA
Age invariance analysis										
Unconstrained	967.59	845	0.002	0.960	0.023					
Measurement weights	982.49	864	0.003	0.962	0.023	14.90	19	0.729	0.002	0.000
Structural covariances	1016.06	885	0.001	0.958	0.024	48.47	40	0.168	−0.002	0.001
Measurement residuals	1057.49	910	0.000	0.952	0.025	89.90	65	0.022	−0.008	0.002
Gender invariance analysis										
Unconstrained	599.74	520	0.009	0.960	0.023					
Measurement weights	624.44	539	0.006	0.962	0.023	24.70	19	0.171	0.002	0.000
Structural covariances	640.19	560	0.010	0.958	0.024	40.45	40	0.450	−0.002	0.001
Measurement residuals	676.84	585	0.005	0.952	0.025	77.10	65	0.145	−0.008	0.002

#### 4.3.7 The reliability of the children’s happiness scale

To assess the reliability of the Children’s Happiness Scale, Cronbach’s α coefficient was calculated. The overall reliability of the Children’s Happiness Scale was found to be 0.91. Moreover, the reliability coefficients for each subscale were as follows: emotionality (α = 0.87), peer relationships (α = 0.88), teacher relationships (α = 0.85), family relationships (α = 0.81), achievement (α = 0.82), and autonomy/freedom (α = 0.81). Therefore, it was confirmed that the final developed Children’s Happiness Scale is a highly reliable tool.

#### 4.3.8 Subfactors and items of the final scale for children’s happiness

The subfactors and items of the final scale for children’s happiness are as follows (see Table 7).

**Table 7:** Composition of subfactors in the final preschoolers' happiness scale.

Subfactor	Content	Item	Number of item
Emotion	Positive, negative emotion	1, 10^*^, 11^*^, 16^*^, 19^*^	5
Peer relationships	Close peer relationships and peer support	2, 3, 17, 18	4
Teacher relationships	Close teacher relationships and teacher support	4, 5, 20, 21^*^	4
Family relationships	Relationship with parents, parental care and love, support from family	7, 12, 13, 25^*^	4
Achievement	Happiness derived from achievement	8, 9, 14, 22	4
Autonomy and freedom	Happiness derived from freedom	6, 15, 23, 24	4
Total			25 items

^*^Items for reverse coding.

## 5 Discussion

Firstly, regarding the discussion on the subscales and measurement method of the happiness scale: Existing happiness scales for Chinese children mainly targeted adolescents and adults. Happiness scales developed for preschoolers had limitations such as measuring only subjective happiness (Wang and Yang, 2013) or being limited to 5-year-old children (Qu, 2018). In this study, we addressed the limitations of existing preschool happiness measurement tools and developed a scale that can measure the happiness of Chinese preschoolers aged 3–5 years. The developed happiness scale comprises six subscales: Emotions, Peer Relationships, Teacher Relationships, Family Relationships, Achievement, and Autonomy and Freedom. This is consistent with previous research that identified emotions (Wang and Yang, 2013; Lee H, 2015; Jung and Shim, 2019; Lee and Kim, 2020) peer relationships (Lee, 2010; Lee and Seo, 2015; Lee H, 2015; Qu, 2018), teacher relationships (Kim and Kim, 2008; Jeon and Lim, 2014; Lee H, 2015; Lee and Kim, 2020) family relationships (Lee, 2010, 2016; Lee and Seo, 2015; Lee H, 2015), achievement (Kim and Kim, 2008; Jeon and Lim, 2014; Qu, 2018), and autonomy and freedom (Qu, 2018) as subfactors of children’s happiness. Furthermore, existing happiness measurement tools for preschoolers primarily used self-reporting by children (Kim and Kim, 2008; Wang and Yang, 2013; Jeon and Lim, 2014; Lee and Seo, 2015; Lee H, 2015; Lee S, 2015; Qu, 2018), teacher ratings (Lee, 2010, 2016; Lee and Seo, 2015), or maternal ratings (Dehghani et al., 2016). In this study, we opted for self-reporting by preschoolers as the measurement method. Happiness is subjective in nature (Diener, 1995), and individuals evaluate their happiness based on their own subjective criteria. Therefore, self-reporting by children is the most appropriate, truthful, and accurate method to assess an individual’s happiness. Secondly, regarding the discussion on the validity and reliability of the happiness scale: At first, through literature review, preschooler interviews, and expert consultations, the subscales and evaluation criteria of the preschooler happiness scale were developed. Subsequently, discriminant validity and item-total correlations confirmed that the scale was composed of distinct and internally consistent items. Exploratory factor analysis revealed that the happiness scale consisted of six subscales and 25 items. Confirmatory factor analysis was conducted to validate construct validity, and all fit indices indicated satisfactory model fit. Additionally, the reliability test of the happiness scale showed significant correlations ranging from 0.81 to 0.91, demonstrating strong internal consistency. In conclusion, this study not only contributes to the theoretical foundation of preschoolers’ happiness but also provides valuable information for the development of future educational programs tailored to promoting happiness among preschoolers. The limitations of this study and suggestions for future research are also discussed. It is hoped that future research will address the limitations identified and further explore various factors that contribute to and maintain preschoolers’ happiness through qualitative research methods.

## 6 Conclusion

This study was conducted to develop a scale measuring happiness in Chinese preschoolers and to validate the reliability and validity of the tool. The conclusions drawn from the results of this study are as follows. Firstly, existing research on happiness scales for preschoolers both domestically and internationally was analyzed, and based on interviews with preschoolers and two rounds of content validity verification by experts, the subfactors and items of the preschooler happiness scale were constructed. Subsequently, a pilot test was conducted to assess the understanding of the happiness scale items among 15 Chinese preschoolers aged 3–5 years. The main study was then carried out with 269 preschoolers to confirm the validity and reliability of the preschooler happiness scale items. It was confirmed that the items were composed of content with high discrimination and homogeneity through item discrimination analysis and item-total correlation analysis. Exploratory factor analysis revealed that the happiness scale consisted of six subfactors: emotion, peer relationships, teacher relationships, family relationships, achievement, autonomy, and freedom, with a total of 25 items. Confirmatory factor analysis and analysis of invariance by age and gender were conducted to verify construct validity, and all indices met the recommended levels of adequacy. The overall reliability of the preschooler happiness scale was 0.91, and by subfactor, it was 0.87 for emotion, 0.88 for peer relationships, 0.85 for teacher relationships, 0.81 for family relationships, 0.82 for achievement, and 0.81 for autonomy and freedom. Therefore, it was confirmed that the final developed preschooler happiness scale is a highly reliable tool. Happiness in young children is not just a fleeting aspect that affects personal development and social integration, but a crucial factor starting from an early age that continuously influences quality of life and human development into adulthood. Many countries recognize the importance of fostering children’s happiness to promote future generations’ progress and social stability. China, amidst rapid social and economic changes, has shown increasing interest in the growth and education of young children. However, research on happiness among Chinese children remains limited, with few studies focusing on parents or early childhood educators. Existing happiness scales developed for Chinese children have limitations, such as measuring only subjective happiness or assessing only 5-year-olds. In contrast, previous studies in other countries have separately measured psychological happiness, social happiness, and subjective well-being. Moreover, happiness is a highly subjective concept where individual cognitive and evaluative assessments of one’s life impact happiness more than objective living conditions. Therefore, it is crucial to develop integrated happiness assessment tools suitable for young children that reflect their thoughts and perceptions. Recent studies indicate that Chinese children exhibit high levels of autonomy-related happiness but lower levels of autonomy itself. Hence, this study considers cultural factors and children’s home environments in China. Continued research to assess happiness levels among young children, especially in China, should focus on developing reliable and valid scales that can objectively and accurately measure children’s happiness.

## Data availability statement

The raw data supporting the conclusions of this article will be made available by the authors, without undue reservation.

## Ethics statement

The studies involving humans were approved by the ethics committee of Shinhan University. The studies were conducted in accordance with the local legislation and institutional requirements. Written informed consent for participation in this study was provided by the participants’ legal guardians/next of kin.

## Author contributions

JX: Writing – original draft, Conceptualization, Data curation, Formal analysis, Investigation, Methodology, Validation. JC: Writing – review & editing, Conceptualization, Supervision.
